# The progress of clinical research on the detection of 1,5-anhydroglucitol in diabetes and its complications

**DOI:** 10.3389/fendo.2024.1383483

**Published:** 2024-05-13

**Authors:** Huijuan Xu, Junhua Pan, Qiu Chen

**Affiliations:** ^1^ Hospital of Chengdu University of Traditional Chinese Medicine, Chengdu, Sichuan, China; ^2^ School of Clinical Medicine, Chengdu University of Traditional Chinese Medicine, Chengdu, Sichuan, China

**Keywords:** blood glucose monitoring, diabetes, screen for diabetes, diabetes management, diabetes complications, 1,5-Anhydroglucitol

## Abstract

1,5-Anhydroglucitol (1,5-AG) is sensitive to short-term glucose fluctuations and postprandial hyperglycemia, which has great potential in the clinical application of diabetes as a nontraditional blood glucose monitoring indicator. A large number of studies have found that 1,5-AG can be used to screen for diabetes, manage diabetes, and predict the perils of diabetes complications (diabetic nephropathy, diabetic cardiovascular disease, diabetic retinopathy, diabetic pregnancy complications, diabetic peripheral neuropathy, etc.). Additionally, 1,5-AG and β cells are also associated with each other. As a noninvasive blood glucose monitoring indicator, salivary 1,5-AG has much more benefit for clinical application; however, it cannot be ignored that its detection methods are not perfect. Thus, a considerable stack of research is still needed to establish an accurate and simple enzyme assay for the detection of salivary 1,5-AG. More clinical studies will also be required in the future to confirm the normal reference range of 1,5-AG and its role in diabetes complications to further enhance the blood glucose monitoring system for diabetes.

## 1 Introduction

In recent years, diabetes prevalence in China has been soaring up in a remarkable way. Poor glycemic control and huge fluctuations in blood glucose over time can lead to complications and influence the prognosis of the heart, kidney, and brain together with other important target organs. Traditional indicators for diagnosing diabetes and monitoring blood glucose fluctuations include fasting plasma glucose (FPG), oral glucose tolerance test (OGTT), glycosylated hemoglobin (HbA1c), and glycated albumin (GA) (1). Nevertheless, FPG is unable to screen patients with isolated postprandial hyperglycemia; the OGTT2h blood glucose detection is complicated to operate, let alone patient compliance; HbA1c is highly susceptible to red blood cell lifespan (2); and HbA1c and GA cannot reflect short-term blood glucose fluctuations. Above all, traditional blood glucose monitoring indicators still have limitations in screening for diabetes and reflecting blood glucose fluctuations. 1,5-Anhydroglucitol (1,5-AG), a nontraditional blood glucose monitoring indicator, is prominently correlated with HbA1c and GA (3). Guidelines for the prevention and treatment of type 2 diabetes mellitus in China (2020 edition) also claimed that 1,5-AG is an auxiliary test indicator for blood glucose monitoring, diabetes screening, and guidance adjustment of therapeutic regimen; it can clearly reflect data from the previous 1 to 2 weeks and postprandial blood glucose fluctuations clearly (1).

Recently, there has been extensive research on the role of 1,5-AG in diabetes and its complications. At the same time, salivary 1,5-AG has also been extensively explored in clinical practice owing to its characteristic of being woundless and its simplicity (**Table 1**). This article reviews the progress of clinical research on the detection of 1,5-AG in diabetes and its complications.

**Table 1:** Advantages and limitations of different screening tests.

Screening test	Advantages	Limitations
Fasting plasma glucose	Inexpensive Convenient Fast	Susceptible to lifestyle influences Requires fasting blood Cannot screen for isolated postprandial hyperglycemia
Oral glucose tolerance test	High diagnostic accuracy	Requires fasting blood Cumbersome operation Poor patient cooperation
Glycosylated hemoglobin	Reflects long-term blood glucose control Highly stable and less affected by lifestyle and food Dose not require fasting blood	Affected by red blood cell life span Does not reflect short-term blood glucose fluctuations
Glycated albumin	Reflects short- to medium-term blood glucose control Not affected by red blood cell life span	Affected by white blood cell renewal rate Cannot check patients with cirrhosis and nephrotic syndrome Affected by body fat content and thyroid hormones
1,5-anhydroglucitol	Reflects short-term blood glucose fluctuations Reflects postprandial blood glucose fluctuations Identification of diabetes subtypes Salivary 1,5-anhydroglucitol is noninvasive and convenient	Affected by many factors The detection method and the normal reference value range are not uniform

## 2 Overview of 1,5-AG

### 2.1 Characteristics and metabolism of 1,5-AG

**Figure 1:**
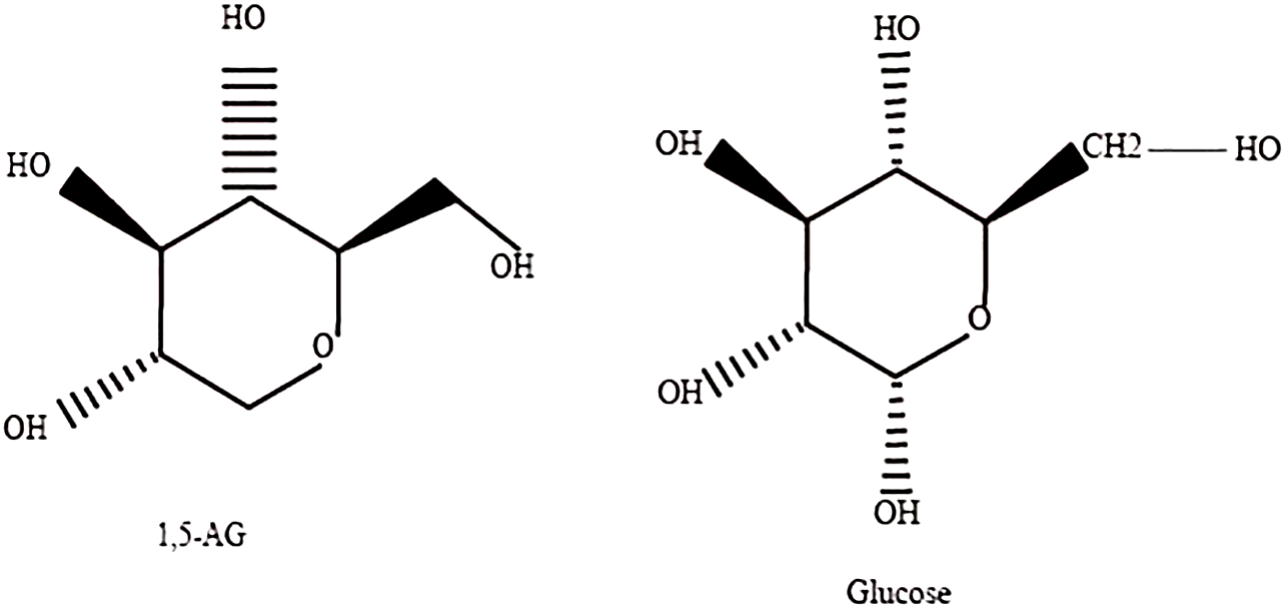
Structure of 1,5-AG and glucose.

**Figure 2:**
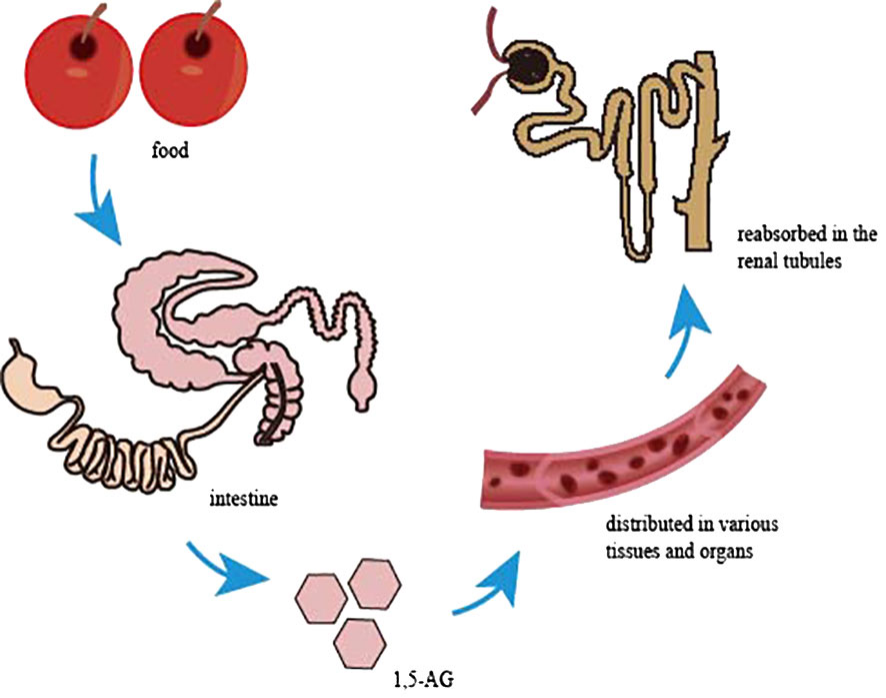
Circulatory pathway of 1,5-AG in the body.

1,5-AG, a naturally occurring, chemically inert monosaccharide with a structure similar to glucose that is mainly derived from food, is absorbed in the intestine and widely distributed in various tissues and organs in free form, with little metabolic degradation in the body (**Figure 1**). Almost all 1,5-AG excreted in urine is reabsorbed in the renal tubules by specific sodium-glucose cotransporter 4 or specific sodium-glucose cotransporter 5 (SGLT4 or SGLT5), and this process (4) is competitively inhibited by glucose since 1,5-AG and glucose share the transporter protein (**Figure 2**). Previous research had suggested that SGLT4 is the renal transporter for 1,5-AG, but more recently it has been demonstrated that 1,5-AG is transported by SGLT5, while mannose is transformed by SGTL4 (5, 6). Nevertheless, there could be some optional bias because these studies are focused on patients with neutropenia. Although the accuracy of this finding is still uncertain, it also provides a new direction for future research. We look forward to more multicenter, large-sample randomized controlled trials in the future to reveal the metabolic mechanisms of 1,5-AG *in vivo*. When the blood glucose exceeds the threshold of the renal glucose (8.9–10.0 mmol/L), 1,5-AG will excrete in large quantities in the urine while reabsorption decreases, resulting in a conspicuous decrease in serum 1,5-AG (7). L Ying et al. (8) found that the metabolic rate of 1,5-AG in hepatocytes and skeletal muscle cells was less than 3%, indicating that 1,5-AG was stable in the body. Meanwhile, 1,5-AG can be freely transported both in and out of the cell in accordance with the concentration gradient to achieve dynamic equilibrium, which exactly shows that 1,5-AG can serve as a biomarker of glucose fluctuations.

**Figure 3:**
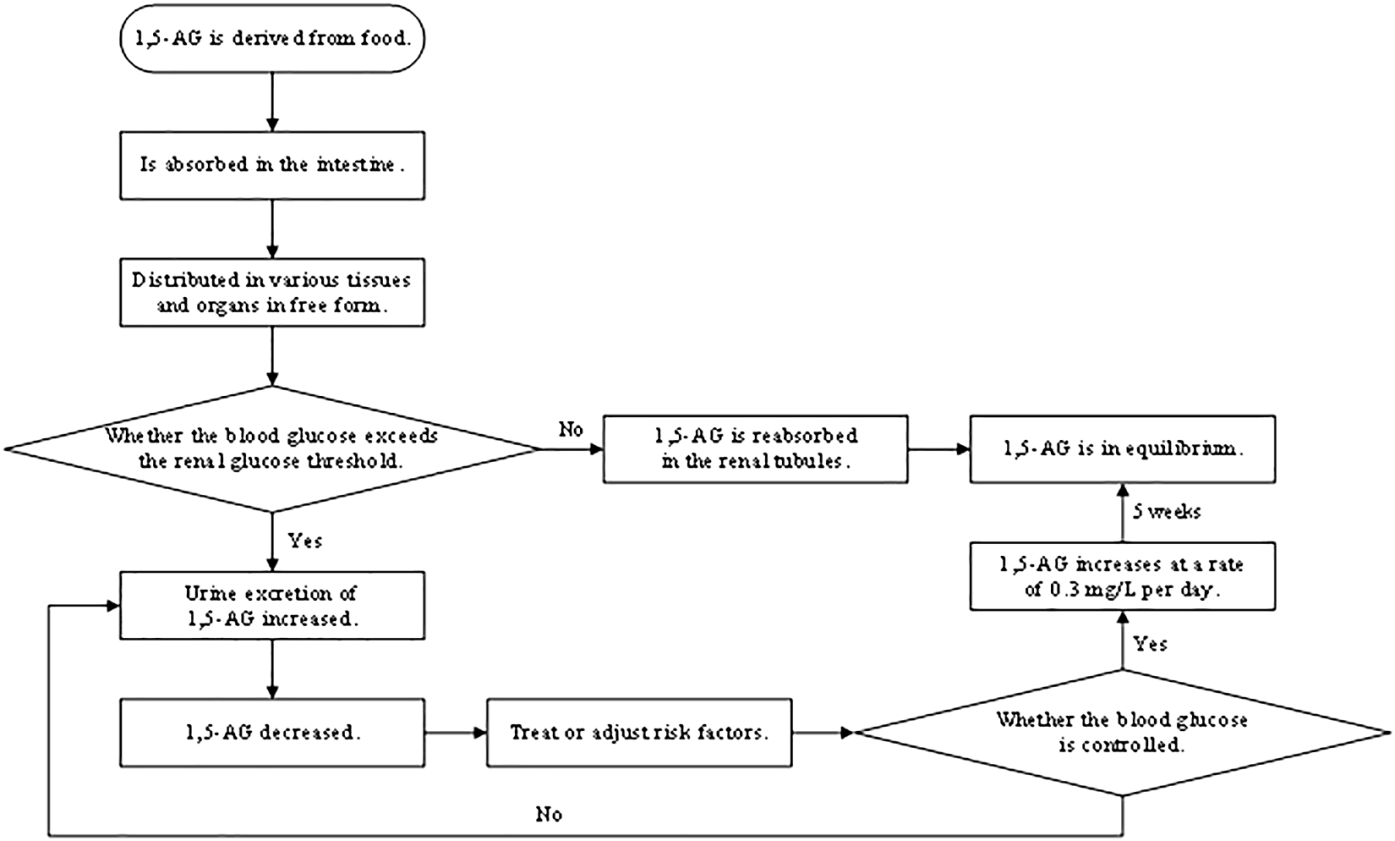
Principle of detection of 1,5-AG.

1,5-AG is sensitive to the reaction, with a half-life of about 1 to 2 weeks, and when blood glucose is controlled, serum 1,5-AG will accordingly increase at a rate of 0.3 mg/L per day, reaching equilibrium after 5 weeks (9). Therefore, 1,5-AG not only reflects blood glucose fluctuations but also records the duration of hyperglycemia. The mass of evidence (10) also indicates that 1,5-AG, a good indicator to monitor short-term blood glucose, is qualified to reflect short-term glucose fluctuations, postprandial hyperglycemia, and even daily glucose excursions (**Figure 3**). JB McGill et al. (11) found that there was a strong correlation between changes in 1,5-AG within 5 days and changes in HbA1c over the subsequent 3 months. Therefore, 1,5-AG could be used as an intermediate marker between 3-month assessments of HbA1c to determine whether glycemic control is good or not for longitudinal monitoring. Because 1,5-AG is dynamic, monitoring glycemic control with 1,5-AG should not rely on a single-point measurement. We believe that in clinical applications, serum 1,5-AG should be tested once at the patient’s initial treatment to assess the patient’s glycemic control and then adopt an appropriate hypoglycemic program. During the patient’s secondary evaluation (such as 1 week later), serum 1,5-AG should be tested again to observe its fluctuation and to assess whether the hypoglycemic program is effective or not based on the degree of relief of the patient’s clinical symptoms and whether the blood glucose level has improved. After a comprehensive evaluation, the clinician will decide whether to change the hypoglycemic program and the timing of the next serum 1,5-AG test so as to realize the individualized approach. Therefore, the timing of serum 1,5-AG testing depends on the duration of the hypoglycemic program adopted by the clinician and the overall judgment of the patient’s condition. As shown in a clinical trial in the United States (12), 1,5-AG levels can sensitively and rapidly reflect glycemic changes after adjustments to personalized treatment strategies, including changes in drug type or dosage, as well as the initiation of insulin therapy or combinations of different insulin regimens. This confirms our proposal to tailor the treatment to the individual by reflecting glucose fluctuations dynamically for longitudinal monitoring.

### 2.2 Detection methods for 1,5-AG

1,5-AG can be detected in a variety of samples such as serum, saliva, and cerebrospinal fluid, in which detection methods have undergone several improvements, now mainly divided into mass spectrometry and enzyme assay two major categories (**Table 2**). Gas chromatography/mass spectrometry (13) and ultra-performance liquid chromatography tandem mass spectrometry (14) are often used to detect 1,5-AG, and such methods have good sensitivity and high accuracy, yet the process is more cumbersome, making it rarely used in the clinical detection of serum 1,5-AG but often used in the detection of saliva and other samples with a smaller content of 1,5-AG. Halama et al. (15) found a significant positive correlation between the results of serum 1,5-AG using enzyme assay and mass spectrometry, indicating that enzyme assay is also very accurate for serum 1,5-AG. GlycoMark™ (GlycoMark, Inc., USA) and Determiner-L (Kyowa Medex, Japan) are the most commonly used enzymatic assay kits in the clinical detection (16). Both can automate and quantify the detection of serum 1,5-AG with high specificity, simple operation, and wide use. The reference ranges of serum 1,5-AG and associated inter-individual biological variation parameters measured by the GlycoMark™ kit and the Determiner-L kit were essentially the same, with comparable results. However, there were minor differences, possibly due to calibration differences between the two kits. Using the GlycoMark™ kit to measure serum 1,5-AG has a good correlation with the rate of blood glucose excursions within a day. This provides a better reflection of short-term blood glucose fluctuations, helps monitor glycemic control, and is simple for clinical application (11). The GlycoMark™ kit for serum 1,5-AG is only affected by glucose, while other monosaccharides are less affected. The freeze–thaw sample has little effect on the GlycoMark™ kit results, which is better than the Determiner-L kit. W Nowatzke et al. (17) concluded that the GlycoMark™ kit is more stable and less susceptible to interference; so, it was more commonly used in the United States to monitor medium-term glycemic control. However, the Determiner-L kit has been used for a long time (16), its detection is more stable, and people recognize it more; so, it is more widely used. Meanwhile, the Determiner-L kit has the unique advantage of a detection limit of 1.0 μg/mL for 1,5-AG (18). Therefore, the exact enzymatic assay kit to be used depends largely on the intent of the person sending the test.

**Table 2:** Different methods to detect 1,5-AG.

Detection methods	Representative methods	Advantages	Limitations
Mass spectrometry	Gas chromatography/mass spectrometry Ultra performance liquid chromatography tandem mass spectrometry	Good sensitivity High accuracy	Cumbersome process High cost
Enzyme assay	GlycoMark™ Determiner-L	Convenient Highly specific	Lack of mature enzymatic assay kit

Whereas the use of saliva samples for diabetes testing could facilitate diabetes screening in public places, there is no well-established enzymatic assay kit for salivary 1,5-AG. Saliva was found to be a stable substrate for biochemical assays by the GlycoMark™ assay kit for the detection of 1,5-AG in serum and saliva samples, respectively. However, the GlycoMark™ kit measured salivary 1,5-AG under the influence of galactose, resulting in a readout that does not correlate with salivary 1,5-AG values as measured by mass spectrometry (15). CH Jian et al. (19) discovered no significant correlation between the enzyme assay for salivary 1,5-AG and serum 1,5-AG. Therefore, the reliability of the enzyme assay for the determination of salivary 1,5-AG and its detection methods still need a lot of experimental research and improvement before they can be applied in clinical practice. In the future, we may try to improve the assay by adding the step of removing galactose before sample testing, which will increase the specificity of the Glycomark™ kit for the determination of salivary 1,5-AG and will be favorable for clinical application. The detection of urine samples is also worth exploring, as a significant correlation between glucose and 1,5-AG concentrations in urine has been reported (20). N Namba et al. (21) compared the serum and urinary 1,5-AG levels in 15 patients with insulin-dependent diabetes mellitus as well as in control subjects. They found that urinary glucose concentrations correlated linearly with the ratio of serum and urinary 1,5-AG concentrations. J Ren et al. (22) developed and validated a rapid ultra-performance liquid chromatography–tandem mass spectrometry method for the detection of urinary 1,5-AG, which is simple, efficient, sensitive, and robust. In summary, the detection of 1,5-AG in saliva and urine specimens expands the diversity of samples, and the diversity of the delivered indicators provides more possibilities for our clinical choices.

Over the years, researchers have continuously explored and updated 1,5-AG detection methods that are more suitable for the clinical diagnosis and management of diabetes. Z Zhou et al. (23) adopted a quantitative study for the colorimetric detection of serum 1,5-AG based on graphene quantum dots and enzyme-catalyzed reactions. Their findings indicated a linear correlation between the absorbance and the concentration of serum 1,5-AG in the range of 20.0–100.0 μg/mL, with a detection limit of approximately 0.144 μg/mL. This method is highly accurate, easy to perform, and inexpensive. The paper-based sensor directly measures serum 1,5-AG in just one step within 10 min, which reduces the effect of excessive glucose in serum samples on the test results with high accuracy and a 1,5-AG detection limit of approximately 3.2 μg/mL (24). The nanozyme-mediated cascade reaction system for the electrochemical detection of serum 1,5-AG has high specificity, sensitivity, stability, and reproducibility. It is also low-cost and easy to construct. Through quantitative studies, G Li et al. (25) found that the peak current of the electrochemical biosensor has a good linear relationship within the serum 1,5-AG concentration range of 0.1–2.0 mg/mL, with a detection limit of approximately 38.2 μg/mL. A novel light-addressable potentiometric sensor can detect serum 1,5-AG with high sensitivity, good recovery, and stability, making it suitable for routine detection. Quantitative studies showed that the potential shift of the light-addressable potentiometric sensor has a linear relationship at a serum 1,5-AG concentration of 10 μg/mL, with a detection limit of approximately 10 μg/mL (26).

### 2.3 Normal reference range of 1,5-AG and its influencing factors

Influenced by age, gender, race, regional environment, diet, and medication, to name just a few, there exist some differences in the normal reference range of serum 1,5-AG. M Welter et al. (27) studied 2,303 healthy subjects of different genders and ages, finding that there was a difference in their 1,5-AG reference range, which was consistent with the results of E Selvin (28). Chen et al. (29) conducted an OGTT test on 646 healthy subjects in Jiangsu Province, which showed that the reference values of 1,5-AG differed by gender, 15.8–52.6 μg/mL in male patients and 14.3–48.0 μg/mL in female patients. The study concluded that 1,5-AG was influenced by factors such as gender, age, and uric acid, which was also significantly lower in blacks than in whites, and its ability to predict complications was also different (30). Serum 1,5-AG concentrations are also affected by types and quantities of dietary carbohydrates (31), excessive intake of dairy products (32), differences in renal glucose threshold (4), and polygala Chinese herbs (33). For the treatment of diabetes, sodium-glucose cotransporter 2 inhibitors, such as dapagliflozin, inhibits renal glucose reabsorption, which can also indirectly inhibit the reabsorption of 1,5-AG by SGLT4 or SGLT5, leading to a decrease in 1,5-AG (34). S Li et al. (35) found that the manufacturer’s reference range of the 1,5-AG kit (>14 μg/mL) was not applicable, and the study results suggested that the reference values of 1,5-AG for male patients in Guangdong Province were 34.61–37.37 μg/mL, compared with 22.38–25.07 μg/mL for female patients.

Compared with blood, saliva is easier to collect and store, and the detection of salivary 1,5-AG is noninvasive, making this indicator work to clinical application. DO Mook-Kanamori et al. (36) found that salivary 1,5-AG was highly correlated with serum 1,5-AG and with blood glucose, HbA1c was negative, through a case–control study of type 2 diabetes mellitus (T2DM), indicating that salivary 1,5-AG can be used to screen for diabetes. CH Jian et al. (37) suggested collecting saliva samples by chewing cotton swabs 40–50 times in 1 min and storing at normal temperature or 4°C for a short period of time. The normal reference range of salivary 1,5-AG measured by liquid chromatography mass spectrometry was 0.09–1.63 mg/L (38).

## 3 Progress of clinical research on 1,5-AG for diabetes

### 3.1 Role of 1,5-AG in screening for diabetes

Most diabetes cases are very insidious at the outset, making the rate of missed diagnoses high, and some of the commonly used blood glucose detection indicators have advantages and disadvantages, while 1,5-AG has a unique advantage in reflecting short-term glucose fluctuations and postprandial hyperglycemia, which results in 1,5-AG detection being gradually applied in clinical practice. As early as the 1980s, some scholars (39) have proposed that the decrease in 1,5-AG level was closely related to diabetes, which could be used as a biomarker of hyperglycemia for screening diabetes. Y Wang et al. (40) conducted an OGTT test on 1,170 subjects, measuring indicators of 1,5-AG, HbA1c, FPG, and 2-h postprandial plasma glucose, respectively. The results showed that serum 1,5-AG level was significantly negatively associated with FPG, 2-h postprandial plasma glucose, and HbA1c and that the optimal cutoff value of 1,5-AG for the diagnosis of diabetes was 11.18 μg/mL, with a sensitivity of 92.6% higher than HbA1c (82.3%) and an area under the curve of 0.920 higher than HbA1c (0.887). T Yamanouchi (41) divided 1,620 subjects into non-diabetic group, impaired glucose tolerance group, diabetes group, and other diseases without impaired glucose tolerance group, with indicators such as 1,5-AG and HbA1c, respectively, and found that the overlap of 1,5-AG values in the four groups was less than those of other indicators, whose reduction was highly specific (93.1%) and sensitive (84.2%) for the diagnosis of diabetes, with an optimal cutoff value of 14 μg/mL.

However, using only 1,5-AG as a biomarker in screening for diabetes has no obvious advantages compared with traditional blood glucose indicators such as HbA1c, GA, and FPG. Combining 1,5-AG with HbA1c, FPG, and GA to screen for diabetes with high sensitivity and specificity is more conducive to clinical application. J Qian et al. (42) conducted a study of 2,184 people in Jiangsu Province which showed that the optimal threshold for 1,5-AG screening for diabetes was ≤23.0 μg/mL, and the sensitivity of HbA1c combined with 1,5-AG was 85% higher than HbA1c (70%). H Su et al. (43) discovered that the sensitivity of 1,5-AG combined with FPG screening for diabetes was 84.92% and the specificity was 91.45% higher than the GA combined with FPG (77.71% and 90.88%) or using the above-mentioned indicators alone. Therefore, 1,5-AG combined with HbA1c, FPG, and GA screening for diabetes can reduce the proportion of people who need OGTT. Moreover, salivary 1,5-AG can also screen for diabetes. C Jian et al. (44) studied 363 people at risk of diabetes and 278 healthy subjects in Shanghai showing that the optimal cutoff value of salivary 1,5-AG was 0.44 μg/mL, and the sensitivity of salivary 1,5-AG combined with HbA1c or FPG was 80.13% and 73.51%, respectively, significantly higher than the above-mentioned indicators alone, which improved the screening rate of diabetes and reduced the proportion of the population requiring OGTT by 51.41%. However, Loomis et al. (45) found that a gene in the human body (SLC5A10) affects the 1,5-AG levels. As a result, 1,5-AG cannot be used as a hyperglycemic biomarker in the case of genetic variations. In the future, more studies are needed to determine whether 1,5-AG can be an effective biomarker for hyperglycemia.

Serum 1,5-AG is applicable to screen for type 1 diabetes mellitus (T1DM), as an auxiliary diagnostic indicator for T1DM (46), and to identify T2DM and fulminant type 1 diabetes mellitus (FT1DM). FT1DM has an acute onset and rapid progression. If it is not diagnosed and treated in time, it will lead to various complications and even death; so, early identification and diagnosis of FT1DM is extremely important. A Pal (47) and M Koga (48) found that the average value of serum 1,5-AG in FT1DM was 3.09 µg/mL, lower than T2DM (5.43 µg/mL), while HbA1c had no significant difference; so, 1,5-AG was more suitable to identify FT1DM and T2DM. L Ying et al. (49) studied 226 subjects with HbA1c <8.7%, showing that the 1,5-AG/GA index contributed to the early identification of FT1DM and the new-onset type 1A diabetes, with an optimal cutoff value of 0.3, which when combined with HbA1c resulted in an improvement of the identification rate of 61.11%. However, the aforementioned studies are not enough. Thus, clinical studies are urgently needed to verify this conclusion.

### 3.2 Role of 1,5-AG in diabetes management

Blood glucose monitoring is especially important for diabetes management. Dynamic monitoring of blood glucose fluctuations can reflect the patient’s glycemic control, identify the risk of diabetes as early as possible, effectively prevent diabetes recurrence, prevent the occurrence of hypoglycemic events, and also serve as a basis for adjusting clinical medication. The familiar continuous glucose monitoring (CGM), which measures the glucose concentration in interstitial fluid using a skin sensor, can provide detailed information about glucose variability. The range of target glucose levels (3.9–10.0 mmol/L) is close to the renal glucose threshold, which reflects glucose fluctuations in a sustained manner, making it an effective monitoring indicator (50). However, CGM readings may be influenced by periods of hyperglycemic variability as well as paracetamol or ascorbic acid intake, and factors such as skin pigmentation and room temperature may also contribute to differences in readings (51). Studies have demonstrated that CGM can continuously record blood glucose levels, which is beneficial in assessing blood glucose fluctuations in T1DM. Unfortunately, CGM is both expensive and inconvenient. The patients’ daily lives may be disturbed and uncomfortable as a result of having to keep the needle immobilized for several days (52). In summary, CGM is not commonly used in clinical practice. Undoubtedly, 1,5-AG presented in this article can be sensitive to reflect short-term blood glucose fluctuations, not affected by mild or moderate renal insufficiency, which is a reliable indicator of glycemic control in T2DM with normal renal function and mild to moderate renal insufficiency (53), and can also be used as an early predictive indicator for T1DM progress (54). A large number of studies (18, 55) have found that changes in 1,5-AG levels are significantly correlated with changes in many CGM variation indicators, indicating that both 1,5-AG and CGM are sensitive to blood glucose fluctuations. However, there is a fundamental difference between 1,5-AG, which uses venous blood, and CGM, which measures glucose concentration in interstitial fluid. There is no doubt that testing venous blood is more conducive to visualizing blood glucose fluctuations. Since CGM requires the wearing of a skin sensor, this is very noticeable and may cause anxiety in some patients. Meanwhile, for patients with insulin pumps, monitoring glucose fluctuations with CGM requires wearing two machines at the same time, which is very inconvenient, whereas testing serum 1,5-AG does not have this disadvantage. The differences between the CGM reading and the blood glucose concentration are approximately 0.55–1.11 mmol/L, with a 10- to 15-min lag time. Serum 1,5-AG, on the other hand, does not experience a delay and therefore responds more rapidly, allowing for better management of diabetic patients (56). It is because serum 1,5-AG is more responsive that its application is more meaningful for severe patients, and the detection of serum 1,5-AG can avoid the occurrence of diabetic critical illnesses such as severe hypoglycemia (45). Meanwhile, 1,5-AG can be detected using existing enzymatic kits, making clinical application easier. J Peabody et al. (57) found that the use of 1,5-AG in clinical practice could improve the quality of preliminary healthcare, better identify patients with poor glycemic control, and reduce the cost of the healthcare system.

BA Kappel et al. (58) confirmed that serum 1,5-AG is the most reliable predictive indicator of poor glycemic control through a comprehensive metabolomics study. J Lin (59) found that 1,5-AG could accurately detect the nuances in blood glucose, rapidly increasing after glycemic control in diabetic patients, and better assess the risk of diabetes. H Sone et al. (60) studied 22 hospitalized T2DM patients who had been educated on diabetes management and then followed up for 3 months after leaving the hospital to measure their 1,5-AG, HbA1c, BMI, and other indicators. The results found that patients with low 1,5-AG had a higher BMI and a higher risk of disease recurrence, while the reflection of HbA1c was not sensitive; so 1,5-AG is more conducive to identify patients with poor glycemic control, and monitoring 1,5-AG levels can effectively prevent diabetes recurrence. Salivary 1,5-AG also plays an important role in predicting the risk of diabetes. Kedarnath et al. (61) discovered that the sensitivity and specificity of 1,5-AG <0.054 µg/mL for predicting blood glucose >180 mg/dl were 86.4% and 87.2%, respectively.

The monitoring of 1,5-AG is also effective in preventing the occurrence of hypoglycemic events, and the lower the 1,5-AG level the greater the risk of developing severe hypoglycemia. MK Kim et al. (62) recruited 18 patients with T2DM treated with insulin, and the results showed a significant negative correlation between 1,5-AG and hypoglycemia score (*r* = -0.510, *P* = 0.031), which remained after adjusting a series of indicators (*r* = -0.468, *P* = 0.068). AK Lee et al. (63) examined a series of biomarkers and assessed the association of risk factors with severe hypoglycemia using the Cox proportional risk regression model in 1,206 diabetic patients at risk of atherosclerosis in the community, which showed a linear correlation between 1,5-AG levels and severe hypoglycemia. Thus, 1,5-AG can be used as a reliable indicator for predicting hypoglycemic events in diabetic patients.

It has also been shown that 1,5-AG can be used as a basis for adjusting the clinical medication of diabetic patients. A clinical trial in the United States (12) found that 1,5-AG levels can sensitively and rapidly reflect glycemic changes after adjustments to personalized treatment strategies, including changes in drug type or dosage as well as the initiation of insulin therapy or combinations of different insulin regimens. KM Dungan et al. (64) discovered that the combination of 1,5-AG and HbA1c may be a reliable indicator for initiating insulin therapy in T2DM patients with poor control of oral hypoglycemic agents in controlled experiments, but the optimal threshold is still unclear and needs to be further explored.

### 3.3 Relationship between 1,5-AG and pancreatic β cells

The main pathogenesis of T2DM involves β cell dysfunction and insulin resistance. In order to better manage diabetes, we should use appropriate methods to detect the β cell function status and the secretion of insulin. In a study of 302 newly diagnosed T2DM patients, X Ma et al. (65) found that 1,5-AG was associated with basal insulin sensitivity and secretion as well as the early insulin secretion of the newly diagnosed T2DM in China. A reduction in the level of 1,5-AG means a decrease in insulin secretion capacity and also reflects a decrease in insulin production index. C Jiménez-Sánchez et al. (66) believed that serum 1,5-AG concentration was closely correlated with the content of β cells, while other glycemic control indicators cannot monitor the loss of β cells; so, for people at a high risk of diabetes, attention should be paid to monitoring serum 1,5-AG to further identify the loss of β cells and monitor the progress of the patient’s condition. In addition to reflecting the content of β cells, 1,5-AG can also reflect its functional status. By comparing lean β-Phb2-/- mouse models and obese db/db mouse models, L Li et al. (67) found that 1,5-AG, a blood glucose biomarker reflecting the degree of β cell function, was closely associated with the decline of functional β cells before the onset of diabetes. Y Shen et al. (68) investigated the relationship between acute C peptide response to arginine and serum 1,5-AG in 623 T2DM patients, showing a linear relationship between the two, while acute C peptide response was an indicator of responsive β cell function, further illustrating that 1,5-AG was closely related to β cell function. H Su et al. (69) measured the levels of 1,5-AG × HbA1c/100 (AHI) in 3,562 people to evaluate islet function and insulin sensitivity in T2DM patients with different AHI levels. The results showed that the normal population had an AHI level of 1.0 (0.7–1.3), which was significantly higher than the T2DM group of 0.8 (0.5–1.2). Hence, AHI can reflect the changes and functions of pancreatic β cells in blood glucose disorders. The lower the AHI, the more severe the blood glucose disorder and the worse the pancreatic β cell function.

Salivary 1,5-AG is a new noninvasive indicator that reflects early insulin secretion function. L Ying et al. (70), through a study of 284 T2DM patients, found that salivary 1,5-AG was closely correlated with the C-peptide production index at 0–30 min and the ratio of the area under the C-peptide curve to the area under the glucose curve. However, A Morita (71) deemed that 1,5-AG had no significant correlation with insulin secretion function. Whether there is a link between the two remains to be verified.

## 4 Progress of clinical research on 1,5-AG for diabetes complications

### 4.1 Role of 1,5-AG in diabetic nephropathy

L Bernard et al. (72) studied 3,799 people at risk of atherosclerosis in the community and found 1,5-AG to be an early sign of chronic kidney disease, which was inversely correlated with glucose and fructose. H Peng (73) suggested that 1,5-AG decreased with impaired renal function and that a low 1,5-AG level predicted a higher risk of developing diabetic nephropathy and was also significantly correlated with the progression of diabetic nephropathy, which was in accordance with the follow-up results of B Yu (74). E Selvin et al. (75) followed 10,000 people at risk of atherosclerosis in the community for 20 years and found that the risk of developing chronic kidney disease was increased threefold in diabetic patients with 1,5-AG <6 μg/mL, even after adjustment of HbA1c or FPG. N Taya et al. (13) studied 31 T2DM and 30 healthy subjects, compared with the biomarkers before and after treatment using gas chromatography–mass spectrometry. According to the study, the decrease in 1,5-AG and the increase in monosaccharide levels implied poor glycemic control and a significant increase in amino acid levels, which aggravated the kidney burden and increased the risk of diabetic nephropathy. Lower levels of 1,5-AG are associated with the risk of developing end-stage renal disease. When a reduction in serum 1,5-AG level is detected, attention should be paid to screening for kidney damage and timely intervention to avoid irreversible outcomes (76). A study (77) of time in range with dynamic blood glucose monitoring showed a significant positive correlation of 1,5-AG with time in range (*r* = 0.591). In conclusion, 1,5-AG not only helps to predict the risk of developing diabetic nephropathy but also serves as an evaluation indicator of glycemic control.

### 4.2 Role of 1,5-AG in diabetic cardiovascular disease

Postprandial hyperglycemia and blood glucose fluctuations contribute to the development of cardiovascular disease, while 1,5-AG is a reliable indicator of monitoring postprandial hyperglycemia and reflecting short-term blood glucose fluctuations, which can be used to reduce the occurrence of cardiovascular disease in diabetic patients by monitoring the 1,5-AG levels (78, 79). A prospective observational study of 1,5-AG and cardiovascular diseases found that low levels of 1,5-AG (<6.0 µg/mL) were closely related to cardiovascular disease (80), and the lower levels of 1,5-AG indicated the higher mortality of cardiovascular events (81). K Torimoto (82) found that a low 1,5-AG level was associated with vascular endothelial dysfunction, which was a potential marker of vascular endothelial dysfunction. Wada et al. (83) studied 161 patients with cardiovascular disease receiving percutaneous coronary intervention and measured the calcification angle by intravascular ultrasound before intervention to reflect the degree of coronary artery calcification. The results showed that the low-1,5-AG group (<14.0 μg/mL) had a significantly higher calcification angle (144°) than the high-1,5-AG group (≥14.0 μg/mL, 107°). YH Zou et al. (84) divided 160 patients with unstable angina pectoris and HbA1c <7.0% into calcified and non-calcified groups. Then, the serum 1,5-AG and alkaline phosphatase levels were monitored, respectively. The results showed that the 1,5-AG levels were significantly decreased and the alkaline phosphatase levels were significantly increased in the calcified group, which also confirmed the correlation between 1,5-AG and coronary artery calcification, and the lower 1,5-AG predicted a higher risk of coronary artery calcification. Serum 1,5-AG also predicts whether coronary plaque ruptures in diabetic patients with acute coronary syndrome (85). On the contrary, B Warren (86) and MR Rooney (87) concluded that 1,5-AG <10 µg/mL was negatively associated with cardiovascular disease, but there was almost no correlation between the two when the value of 1,5-AG was high; so, 1,5-AG has a poor predictive effect on cardiovascular disease, shows distinct limitations, and cannot provide prognostic information on cardiovascular events in diabetic patients.

### 4.3 Role of 1,5-AG in diabetic retinopathy

E Selvin et al. (88) has followed-up patients over 5 years and found that 1, 5-AG was inversely related to microvascular events and mortality, which means that a lower 1,5-AG level significantly increased the incidence of microvascular events, increased patient mortality, and was closely associated with retinopathy. WJ Kim et al. (89) followed 267 T2DM patients for 5 years and discovered that the risk of developing diabetic retinopathy in the low-1,5-AG (<5.1 ug/mL) group was significantly higher than that in the high-1,5-AG (≥8.64 ug/mL) group. A study has also shown that low levels of 1,5-AG were positively correlated with the incidence of diabetic retinopathy, and the prevalence of 1,5-AG <6 µg/mL was 11 times higher than that of 1,5-AG ≥10 µg/mL (75). N Mukai et al. (90) studied 2,681 subjects to locate the optimal threshold for detection of diabetic retinopathy through measurements of 1,5-AG and GA and ophthalmic examinations. According to the study’s results, the optimal thresholds for each indicator were as follows: FPG—6.3 mmol/l and 1,5-AG—12.1 μg/mL. The incidence of diabetic retinopathy was significantly increased when 1,5-AG <12.1 μg/mL, which indicated that monitoring 1,5-AG was essential to prevent microangiopathy and could be widely used in clinical applications.

### 4.4 Role of 1,5-AG in diabetic pregnancy complications

1,5-AG, a hyperglycemia biomarker in pregnant women, serves to be a bad predictor of gestational diabetes. To better confirm this view, scholars have conducted numerous clinical studies (91, 92). TA Pramodkumar et al. (93) measured serum 1,5-AG by recruiting 145 pregnant women without gestational diabetes and 75 pregnant women with gestational diabetes, and the study found that the mean value of 1,5-AG in pregnant women with gestational diabetes was 0.001 ± 16.2 μg/mL, which was significantly lower than that in pregnant women without gestational diabetes (1.5 ± 11.8 μg/mL). 1,5-AG remained significantly correlated with gestational diabetes, even after adjusting the potential confounders. Moreover, 1,5-AG has a unique role in predicting diabetic pregnancy complications. LA Wright et al. (94) compared the relationship between 1,5-AG, HbA1c and diabetic pregnancy complications in 17 gestational diabetes cases, 48 T1DM cases, and 37 T2DM cases. The study found that the level of 1,5-AG was significantly negatively correlated with diabetic pregnancy complications, especially in large-for-gestational-age (LGA) and neonatal hypoglycemia cases. There is a strong positive correlation between 1,5-AG and preeclampsia at 1 week of gestation, which was also supported by E Yefet (95). CL Meek et al. (96) monitored the relationship between the biochemical indicators of T1DM in 157 pregnant women and pregnancy complications and found that 1,5-AG, the most prevalent indicator of LGAs, was negatively correlated with LGA throughout the entire pregnancy, especially in the late trimester of pregnancy, with lower 1,5-AG levels meaning a greater risk of LGA (97). 1,5-AG also predicts growth restriction in full-term fetuses and is closely linked to neonatal mortality (98).

### 4.5 Role of 1,5-AG in diabetic peripheral neuropathy

M Yamawaki et al. (99) examined brain MRI, serum 1,5-AG, and cognitive function in 688 subjects. According to the study’s results, lower 1,5-AG levels were found to be positively correlated with severe periventricular hyperintensities and deep white matter hyperintensities, as well as a significant risk factor for cognitive decline and depression. For every 1,5-AG reduction of 5 μg/mL, the risk of dementia increases by 16%; so, the decrease of 1,5-AG is a risk factor for cognitive decline and dementia, and the monitoring of serum 1,5-AG can be an important method of preventing cognitive decline (100). Q Lou et al. (101) randomly divided 75 patients with T2DM after cerebral infarction into two groups for a 6-month randomized controlled experiment. The control group received conventional treatment, while the intervention group strengthened the monitoring of blood glucose fluctuations on the basis of conventional treatment and flexibly adjusted medication. The results showed a marked improvement of 1,5-AG in the intervention group, and the National Institutes of Health Stroke Scale score was also reduced significantly; so, monitoring 1,5-AG could reduce the damage to the patient’s neurological function and improve the quality of life. However, in recent years, studies have shown that poor glycemic control and a longer course of diabetes were significantly associated with cognitive impairment, which can be reflected through HbA1c, GA, and fructosamine, while 1,5-AG is not significantly related to the occurrence of dementia (102). CW Hicks et al. (103) also concluded that 1,5-AG was not significantly related to diabetic peripheral neuropathy. Therefore, more studies are needed to confirm whether 1,5-AG is associated with diabetic peripheral neuropathy.

## 5 Prospect

Although many clinical studies have demonstrated the value and practicality of 1,5-AG in clinical applications, there are still some problems to think about and study. First, while mass spectrometry detects serum 1,5-AG with high sensitivity and accuracy, the process shows difficulty, inconvenience, and high cost. Enzyme detection is simple and easy to perform, but the results will be affected by the manufacturer and the quality of the kit. Salivary 1,5-AG has been verified to be correlated with serum 1,5-AG, which has attracted much attention due to its noninvasiveness and good application potential. However, mass spectrometry for the detection of salivary 1,5-AG is cumbersome and expensive, and due to the lack of mature enzyme assay kits, the present enzyme assay for salivary 1,5-AG has poor accuracy. Therefore, a large number of studies are still needed in the future to identify the best detection method for 1,5-AG in order to promote its clinical application.

Second, the normal reference range for 1,5-AG is not constant, which is influenced by age, gender, race, regional environment, diet, medication, and so on. Establishing a normal reference range is an essential step in the application of 1,5-AG in clinical practice, but the reference ranges given by many manufacturers are not applicable and vary from region to region. With the multiplication of clinical studies, the normal reference range of 1,5-AG has been proposed in most regions, and more large-scale clinical studies will be needed in the future to further determine the normal reference range in each region.

Third, 1,5-AG is sensitive to short-term blood glucose fluctuations and postprandial hyperglycemia, helping to assist the screening for diabetes and avoiding the missed diagnosis of postprandial hyperglycemia patients. However, 1,5-AG cannot reflect the specific time of poor glycemic control. More research can be done in the future to determine if it can reflect specific periods more conducive to accurate medication administration and glycemic control.

Fourth, 1,5-AG is widely used but is less accurate in patients with liver disease. Increased inositol levels in uremia patients can also interfere with the measurement of 1,5-AG. M Koga et al. (104) showed that serum 1,5-AG levels were lower in patients with chronic liver disease irrespective of their blood glucose levels, which was associated with impaired liver function. The liver produces a modest quantity of 1,5-AG, which is lowered when the liver function is impaired. The liver is an important site of glucagon metabolism and an important organ in the regulation of plasma glucose levels; so, patients with chronic hepatitis, cirrhosis, and other chronic liver diseases often have abnormal glucose metabolism and large fluctuations in blood glucose (105). The reabsorption of 1,5-AG in renal tubules is competitively inhibited by glucose, and when glucose metabolism is abnormal, it will also lead to fluctuations in the measured 1,5-AG value; so, the measurement of serum 1,5-AG in diabetic patients with chronic liver disease does not accurately reflect their glycemic control. Some studies (106) have also shown that 1,5-AG decreases significantly and gradually with the progression of liver fibrosis; so, even if a low serum 1,5-AG level is measured, it does not indicate poor glycemic control. At this time, the use of 1,5-AG to reflect the glycemic control of patients with liver disease is less accurate; so, many studies have concluded that 1,5-AG is not suitable as a glycemic monitoring indicator for patients with liver disease. Therefore, the applicability of 1,5-AG is limited and needs further clinical verification.

Fifth, 1,5-AG is a reliable indicator of poor glycemic control and has a predictive effect on the occurrence of adverse events such as hypoglycemia. However, whether 1,5-AG has a better predictive effect on adverse events than HbA1c, GA, and FPG and other traditional blood glucose indicators remains to be explored. In the future, more prospective studies are required to further clarify whether 1,5-AG has an advantage in predicting adverse events.

Sixth, FT1DM has an acute onset and rapid progression, which can easily pose to various complications and even death; so, early recognition and diagnosis of FT1DM are particularly important. Currently, it is suggested that 1,5-AG can distinguish between FT1DM and T2DM, but such clinical studies are few, not convincing, and may be a small-probability event. A large number of clinical trials will still be needed in the future to confirm this conclusion.

Seventh, 1,5-AG is correlated with pancreatic β cells, but the deeper mechanism remains unclear, and more experimental studies are needed to explore the mechanism of both. Eighth, there are few studies on the role of 1,5-AG in diabetic peripheral neuropathy, and existing studies have different conclusions. Therefore, it is impossible to determine whether 1,5-AG can effectively predict the occurrence of diabetic peripheral neuropathy, and more studies are needed to clarify this. Overall, there are many studies on 1,5-AG with complications, but there is still a lack of prospective cohort studies with large samples, which is not forceful enough. More experimental studies and longer follow-up observations are required to further confirm the clinical value of 1,5-AG for predicting diabetes complications. At the same time, it remains to be demonstrated whether 1,5-AG predicts the risk of diabetes complications better than traditional glycemic indicators such as HbA1c, GA, and FPG.

In summary, 1,5-AG is more sensitive to reflecting short-term glucose fluctuations and postprandial hyperglycemia than traditional glucose monitoring indicators such as HbA1c, FPG, GA, and so on. The combination of 1,5-AG and traditional glucose monitoring indicators improves the accuracy of diabetes screening, which is of great help to improve the glucose monitoring system. 1,5-AG shows great potential in the screening and management of diabetes, diabetes complications, and so on, which is conducive to clinical application. As a noninvasive detection indicator, salivary 1,5-AG is more convenient, but it still requires further research and improvement methods to be widely used. More clinical studies are needed to demonstrate the normal reference range of 1,5-AG and its role in diabetes complications, thus making it better to predict the risk of diabetes complications.

## Author contributions

HX: Conceptualization, Writing – original draft, Writing – review & editing. JP: Writing – original draft, Writing – review & editing. QC: Supervision, Writing – review & editing.
