# Supplementary material for: The progress of clinical research on the detection of 1,5-anhydroglucitol in diabetes and its complications
Source: Front Endocrinol (Lausanne). 2024 May 13;15:1383483. doi: 10.3389/fendo.2024.1383483 (PMC11128578; doi:10.3389/fendo.2024.1383483)
Supplement: Supplementary file 1 [file DataSheet_1.docx]

Supplementary Material

The progress of clinical research on the detection of 1,5-anhydroglucitol in diabetes and its complications

Huijuan Xu 1, Qiu Chen*

*** Correspondence:** Corresponding Author: chenqiu1969@163.com

# Supplementary Figures and Tables

## Supplementary Figures

**
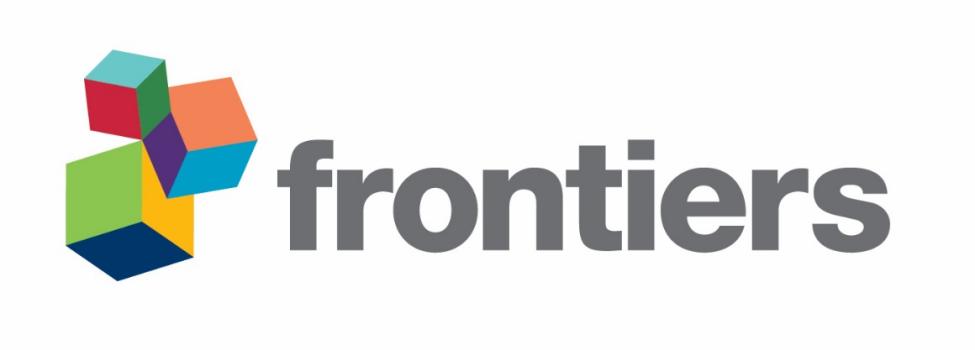
**

**Supplementary Figure 1.** The structure of 1,5-AG and glucose.


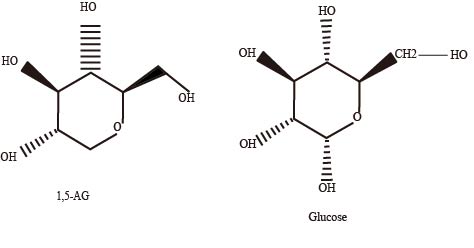


**Supplementary Figure 2.** Circulatory pathway of 1,5-AG in the body.


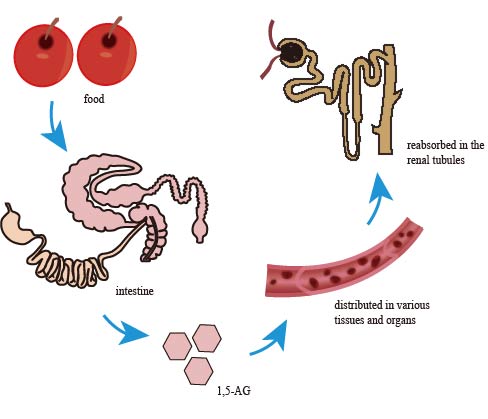


**Supplementary Figure 3.** The principle of detection of 1,5-AG.


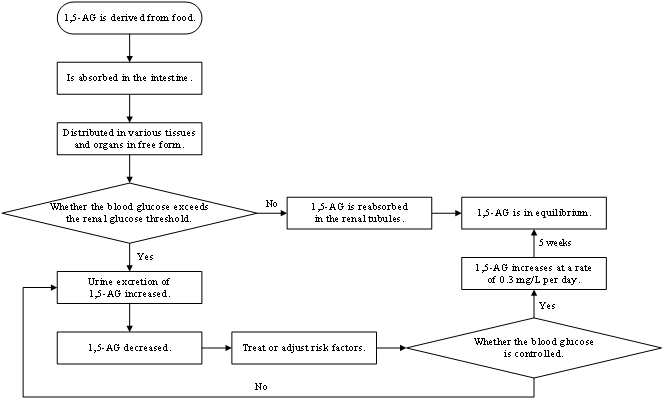


## Supplementary Tables

**Supplementary Table 1.** The advantages and limitations of different screening tests.

| Screening test | Advantages | Limitations |
| --- | --- | --- |
| Fasting Plasma Glucose | Inexpensive.  Convenient.  Fast. | Susceptible to lifestyle influences.  Requires fasting blood.  Cannot screen for isolated postprandial hyperglycemia. |
| Oral Glucose Tolerance Test | High diagnostic accuracy. | Requires fasting blood.  Cumbersome operation.  Poor patient cooperation. |
| Glycosylated Hemoglobin | Reflects long-term blood glucose control.  Highly stable and less affected by lifestyle and food.  Dose not require fasting blood. | Affected by red blood cell life span.  Does not reflect short-term blood glucose fluctuations. |
| Glycated Albumin | Reflects short-to-medium-term blood glucose control.  Not affected by red blood cell life span. | Affected by white blood cell renewal rate.  Cannot check patients with cirrhosis and nephrotic syndrome.  Affected by body fat content, thyroid hormones. |
| 1,5-anhydroglucitol | Reflects short-term blood glucose fluctuations.  Reflects postprandial blood glucose fluctuations.  Identification of diabetes subtypes.  Salivary 1,5-anhydroglucitol is noninvasive and convenient. | Affected by many factors.  The detection method and the normal reference value range are not uniform. |

**Supplementary Table 2.** Different methods to detect 1,5-AG.

| Detection methods | Representative methods | Advantages | Limitations |
| --- | --- | --- | --- |
| Mass spectrometry | Gas chromatography/mass spectrometry.  Ultra performance liquid chromatography tandem mass spectrometry. | Good sensitivity.  High accuracy. | Cumbersome process.  High cost. |
| Enzyme assay | GlycoMark™.  Determiner-L. | Convenient.  Highly specific. | Lack of mature enzymatic assay kit |
